# Attitudes, Beliefs, and Clinical Decision‐Making Regarding Low Back Pain: A Cross‐Sectional Study of Pakistani Medical Practitioners, Physiotherapists, and Exercise Professionals

**DOI:** 10.1002/msc.70228

**Published:** 2026-05-06

**Authors:** Muhammad Naseeb Ullah Khan, Aastha Malhotra, Melainie Cameron

**Affiliations:** ^1^ School of Health, Psychological and Medical Sciences University of Southern Queensland Ipswich Queensland Australia; ^2^ Faculty of Health Southern Cross University Lismore New South Wales Australia

**Keywords:** biopsychosocial model, clinical decision‐making, fear‐avoidance, healthcare professionals, low back pain, pain beliefs, Pakistan

## Abstract

**Background:**

Low back pain (LBP) is a leading global cause of disability, particularly burdensome in LMICs like Pakistan, where biomedical approaches remain dominant in clinical care. This study examined how healthcare professionals' (HCPs) beliefs, attitudes, and confidence influence clinical decision‐making for LBP.

**Methods:**

A cross‐sectional survey of 343 HCPs comprising medical practitioners (*n* = 123), physiotherapists (*n* = 124), and exercise professionals (*n* = 96) in Pakistan was conducted between August 2023 and December 2024. Participants completed the PABS‐PT, Medical Scans Beliefs Questionnaire (MSBQ), two clinical vignettes, and rated their confidence in the use of various LBP treatments. Group differences were tested with ANOVA and chi‐square. Stepwise linear regression and binary logistic regression identified predictors of confidence, decision‐making, and treatment practices.

**Results:**

Physiotherapists demonstrated the strongest biomedical orientation (*p* = 0.01), with no significant group differences in biopsychosocial orientation. MSBQ scores (mean = 17.27) were moderate across all groups, with high agreement that imaging is essential for LBP care. Clinical decisions were predicted by experience, physical activity, gender, and biomedical orientation. Biopsychosocial beliefs predicted greater confidence in pain education, exercise, and CBT (*p* < 0.001) but not in their actual use. Physiotherapists and exercise professionals were significantly more likely than medical practitioners to employ pain education as a treatment strategy (physiotherapists: OR = 5.7, 95% CI = 3.1–10.7; exercise professionals: OR = 6.0, 95% CI = 3.2–11.3; both *p* < 0.001).

**Conclusions:**

Pakistani HCPs show a prevalent biomedical orientation and share some persistent misconceptions about the value of imaging in LBP care. While biopsychosocial beliefs enhance confidence in evidence‐based treatments, translation into practice remains limited. We recommend targeted educational interventions, curricular reforms, and interdisciplinary collaborations to facilitate biopsychosocially informed care.

## 1 Introduction

Low back pain (LBP) is a significant global health concern, contributing substantially to disability‐adjusted life years (DALYs) and imposing a substantial socioeconomic burden on healthcare systems worldwide (M. L. Ferreira et al. 2023; Wu et al. 2020). While efforts to mitigate this burden have advanced in high‐income countries, the situation in low‐ and middle‐income countries (LMICs), including Pakistan, remains challenging due to resource constraints, limited public health education, and a high prevalence of labour‐intensive occupations (Fatoye et al. 2023; Williams et al. 2015; Anema et al. 2009). In Pakistan, where only 1.2% of the GDP is allocated to healthcare (Finance Division and Government of Pakistan 2024; S. J. Khan et al. 2023), access to timely and effective healthcare is limited, particularly in rural and low‐income urban populations. Public health facilities are under‐resourced, and private care, often the only available option, is often expensive and unaffordable (S. U. Khan and Hussain 2020; Sohaib et al. 2025; S. A. Khan 2019; Heltberg and Lund 2009).

Historically, the clinical management of LBP has been grounded in the biomedical model (Buchbinder et al. 2013), which attributes pain and disability solely to structural abnormalities. However, this reductionist approach often leads to over‐emphasis on imaging, medication, and invasive procedures (Maher et al. 2016; Foster et al. 2018). This approach may be effective in some acute or structural conditions. However, it has demonstrated limited long‐term efficacy in managing non‐specific LBP and associated disability (Maher et al. 2016; Foster et al. 2018). A recent systematic review examining medical imaging overuse in LMICs reported that LBP was the most frequent driver of unnecessary scans in more than half of the studies reviewed (Protheroe 2024). In contrast, the biopsychosocial model provides a more comprehensive framework by integrating biological, psychological, and social factors to explain and manage pain, particularly in persistent cases (Zhou et al. 2024). Psychosocial contributors, such as fear‐avoidance beliefs, catastrophizing, and low self‐efficacy, are now recognised to perpetuate disability by limiting patients' physical activity and overall function (Darlow et al. 2011; Foster et al. 2018; M. N. U. Khan et al. 2020).

The prominence of biomedical approaches in LBP care in Pakistan is likely multifactorial (M. N. U. Khan et al. 2025), shaped by both global historical influences and local contextual forces. Colonial medical systems established during British rule promoted pathoanatomical understanding of disease. These systems informed the development of Pakistan's medical education frameworks (Naidu 2023). These legacies persist today in curricula that emphasise diagnosis and pharmacology while providing limited exposure to pain science, behavioural health, or interprofessional education (Javed et al. 2020). Socioculturally, patients often associate visible diagnoses, such as X‐rays or MRIs, with legitimacy, and professionals may feel pressure to conform to expectations for imaging or quick‐fix solutions (Sharma et al. 2020). Moreover, underinvestment in mental health and preventive services contributes to a continued reliance on curative, structurally focused care. These systemic patterns present substantial barriers to integrating biopsychosocial approaches in everyday practice.

Global LBP management guidelines increasingly advocate for the biopsychosocial approach, emphasising active rehabilitation strategies, including exercise therapy, patient pain education, and self‐management (De Campos 2017; Oliveira et al. 2018). Yet even in high‐income contexts, HCPs often struggle to apply these recommendations consistently (Zadro et al. 2019; Buchbinder et al. 2018; Traeger et al. 2019). This gap in implementation may be attributed to persistent beliefs rooted in biomedical thinking, lack of time, practitioners' limited confidence and training in psychosocial aspects of care. Beliefs and attitudes held by HCPs have been shown to influence key clinical decisions such as referral patterns, imaging use, advice about activity, and selection of treatment modalities (Slade et al. 2015; Darlow et al. 2011).

In Pakistan, primary and musculoskeletal healthcare is largely delivered by medical practitioners and physiotherapists, with a growing role for exercise science professionals (H. Y. Khan et al. 2024). These HCPs often work in resource‐constrained environments that may limit their ability to adopt evidence‐based clinical practices (Hanif et al. 2025). Research from other LMICs highlights similar challenges, including cultural preferences for passive treatments (e.g., rest, medication, massage), while placing less emphasis on patient education or psychosocial factors (Morriss and Roques 2018; Peacock and Patel 2008; Sharma et al. 2024). Despite limited evidence from Pakistan regarding HCP behaviour, patient‐level studies demonstrate high rates of fear‐avoidance beliefs, catastrophizing, and psychological distress. These factors mediate pain‐related disability and suggest that psychosocial contributors are inadequately addressed in clinical contexts (M. N. U. Khan et al. 2020).

Although numerous studies have examined the attitudes and beliefs of HCPs in high‐income countries, there are limited data from LMICs like Pakistan. Understanding these attitudes and beliefs is essential, as they influence treatment adherence, patient pain education, and clinical decision‐making. This study aimed to investigate how LBP related attitudes, beliefs, and key professional characteristics (including postgraduate training, clinical experience, confidence in managing LBP, field of practice, and personal physical activity levels) among Pakistani medical practitioners, physiotherapists, and exercise professionals shape their clinical decision‐making and advice for LBP management. The findings aim to inform context‐sensitive strategies to improve LBP care delivery and support the broader integration of biopsychosocial principles in musculoskeletal healthcare in Pakistan and similar settings.

## 2 Material and Methods

### 2.1 Study Design and Participants

This was a cross‐sectional study conducted in Pakistan between August 2023 and December 2024 to examine the attitudes, beliefs, and clinical practices of healthcare professionals (HCPs) towards low back pain (LBP). The target population included medical practitioners, physiotherapists, and exercise science professionals involved in musculoskeletal care.

Inclusion criteria were: Pakistani healthcare professionals who manage people with LBP (medical practitioners, physiotherapists, exercise professionals). HCPs were invited using lists from the Pakistan Medical Association and the Pakistan Physiotherapy Association; exercise professionals were recruited by contacting major health clubs. Additional participants across all groups were invited via social media networking pages of professional groups and local clinics. Responses were excluded if any major survey sections (e.g., belief measures, vignettes, confidence items) were incomplete or missing. A priori sample size estimation was performed using G*Power 3.1 (Faul et al. 2009). To achieve 80% power for a multiple linear regression with four predictors, detecting a small‐to‐moderate effect size (*R* ^2^ = 0.13, *α* = 0.05), a minimum of 85 participants was required. We increased the required sample size by 30% to account for potential model instability linked with stepwise regression, resulting in a minimum sample of > 111. Data were collected using the University of Southern Queensland's online survey tool, which can be accessed and completed on a smart phone, laptop, or desktop computer.

### 2.2 Ethical Considerations

This study was approved by the Human Research Ethics Committee of the University (approval # ETH2023‐0010). Prior to proceeding with data collection, participants provided implied consent by proceeding with and completing the online survey after reading the study information and consent details presented at the beginning of the survey for participation and publication of anonymous data.

### 2.3 Measures

#### 2.3.1 Demographic Characteristics

Collected variables included age, gender, education level, occupation/field of practice (medical practitioners, physiotherapist, exercise professional), years of clinical experience, practice type (public/private), recent experience with LBP, location, personal physical activity level and type of physical activity. Clinicians' personal physical activity was collected as a potential explanatory variable for differences in clinical decision making and confidence in LBP management.

#### 2.3.2 Pain Attitudes and Beliefs

Pain attitudes and beliefs scale for physiotherapists (PABS‐PT) was used to assess attitudes and beliefs of HCPs towards LBP (Houben et al. 2004). The PABS‐PT was initially created for physiotherapists; however, it has been used in studies of general medical practitioners (GPs), osteopaths, and chiropractors (Eland et al. 2018; Bishop et al. 2008; Fitzgerald et al. 2020; Innes et al. 2015). PABS‐PT was selected because it is applicable to the scope of GPs and exercise‐based practitioners (physiotherapists and exercise professionals) and the outcomes can be generalised to the larger body of literature. Each item is scored on a 6‐point Likert scale and has two subscales (10 items relating to biomedical approach and 9 relating to biopsychosocial approach). The score ranges from totally disagree (1) to totally agree. The greater the score in a subscale indicates stronger biomedical or biopsychosocial orientation. The internal consistency of the PABS‐PT biomedical subscale is *α* = 0.80 and the biopsychosocial subscale is *α* = 0.88 and reliability (ICC values) are 0.81 and 0.65, respectively (Mutsaers et al. 2012).

#### 2.3.3 Beliefs About Medical Scans for LBP

Medical scans beliefs questionnaire (MSBQ) was used to evaluate aspects of beliefs of HCPs about the utility of medical imaging for LBP in relation to causation, severity, and treatment response (Beales et al. 2021). Participants were asked to rate each statement on a scale of 1–5, where 1 meant ‘strongly disagree’, 3 meant ‘neutral’ and 5 meant ‘strongly agree’. Item scores were summed to produce a total score (range 5–25), with higher scores indicating stronger beliefs that imaging is necessary, that scan findings explain pain severity, and that imaging is important for guiding management.

#### 2.3.4 Clinical Decision Making

The two vignettes adapted from Rainville et al. (2000) were used in this study. Two case scenarios of hypothetical CLBP patients were presented and participants were asked for their opinion in the form of four questions regarding severity of patient's symptoms, suspected pathology, appropriate activity level and work/return to work advice appropriate for that patient. All vignette items were scored on a Likert scale ranging from 1 to 5. For symptom severity and suspected pathology, higher scores indicated greater perceived severity and greater likelihood of serious structural pathology. For activity and work/return‐to‐work advice, higher scores indicated more restrictive recommendations (i.e., greater limitation of activity and work participation).

#### 2.3.5 Confidence in Treatment Modalities

Participants rated their confidence in the effectiveness of various LBP treatments, including exercise, manual therapy, pain education, cognitive behavioural therapy (CBT), surgery, prescription medications, and over‐the‐counter pain medications. Confidence ratings were captured on a 6‐point scale ranging from ‘total unconfident’ (1) to ‘totally confident’ (6).

### 2.4 Statistical Analysis

All analyses were conducted using SPSS (v 26). Descriptive statistics were calculated for demographic and survey variables, including frequencies, percentages for categorical variables (e.g., gender, professional group), and means, standard deviations, and ranges for continuous variables (e.g., age, years of experience). The reliability of the PABS‐PT and MSBQ was assessed using Cronbach's alpha, with a value of ≥ 0.7 indicating acceptable internal consistency in this sample. Between‐group differences in subscale scores and vignette responses were analysed using one‐way analysis of variance (ANOVA) with the professional group (medical practitioners, physiotherapists, and exercise professionals) as the independent variable. When a significant overall effect was found, post hoc Tukey's HSD tests were conducted to identify pairwise differences. Chi‐square tests were used to explore associations between categorical demographic variables and professional groups. Effect sizes for ANOVA were calculated using eta squared (*η* ^2^), with thresholds of 0.01, 0.06, and 0.14 for small, medium, and large effects, respectively. A *p* < 0.05 was considered statistically significant for all analyses.

Pearson's correlation coefficients were calculated to examine associations between biomedical and biopsychosocial subscale scores. Forward stepwise linear regression analyses were conducted to examine associations between clinician and professional characteristics and PABS‐PT subscale scores on responses to various treatment options (e.g., pain medications, surgery, exercise, pain education) and clinical decision‐making variables from vignettes. Candidate predictors entered stepwise included age, gender, profession, education level, years of clinical experience, further musculoskeletal pain training, personal physical activity intensity, and PABS‐PT biomedical and biopsychosocial subscale scores. Forward stepwise selection used a probability‐of‐*F* entry criterion of *p* < 0.05 and a removal criterion of *p* > 0.10. This approach was used to identify a subset of predictors associated with each outcome from a set of potentially correlated clinician and professional characteristics. Similar variable‐selection strategies have been used in cross‐sectional research examining clinicians' beliefs and practices to identify associations of interest (L. S. Ferreira et al. 2023). Standardised beta coefficients (*β*) and adjusted *R* ^2^ were used to interpret the effect size and model fit. To further investigate treatment‐related behaviours, binary logistic regression analyses were conducted to examine the likelihood of recommending specific interventions (e.g., exercise, pain education) based on profession and belief orientations. Odds ratios (OR) with 95% confidence intervals (CI) were reported. Model fit was evaluated using the omnibus test and Nagelkerke *R* ^2^.

## 3 Results

Table 1 presents the baseline characteristics of the sample (*N* = 343). The final dataset included 123 medical practitioners (35.9%), 124 physiotherapists (36.2%), and 96 exercise professionals (28.0%). The majority of participants were aged 26–30 years (33.2%), with physiotherapists skewing younger (39.5% aged 18–25), and exercise professionals showing a more even age distribution. The gender distribution was nearly equal overall (48.7% female, 51.3% male). However, profession‐specific trends were observed: 70.2% of physiotherapists were female, while 69.8% of exercise professionals were male.

**TABLE 1:** Demographic and professional characteristics of study participants.

Characteristics	Overall (*n* = 343)	Medical practitioners (*n* = 123)	Physiotherapists (*n* = 124)	Exercise professionals (*n* = 96)
Age				
18–25 years	87 (25.4%)	18	49	20
26–30 years	114 (33.2%)	56	38	20
31–35 years	66 (19.2%)	28	17	21
36–40 years	47 (13.7%)	9	13	25
40–45 years	16 (4.7%)	5	4	7
46 years +	13 (3.8%)	7	3	3
Gender				
Female	167 (48.7%)	51	87	29
Male	176 (51.3%)	72	37	67
Education				
Bachelor's degree (16 years)	41 (12%)	5	3	33
Bachelor's + masters (16 years)	48 (14%)	—	3	45
DPT	103 (30%)	—	103	—
MBBS	85 (24.8%)	85	—	—
M.Phil./M.S/t‐DPT	19 (5.5%)	1	11	7
Post grad/doctorate	47 (13.7%)	35	1	11
Experience				
Less than 2 years	82 (23.9%)	23	41	18
2–5 years	115 (33.5%)	44	50	21
5–10 years	82 (23.9%)	34	22	26
11–20 years	53 (15.5%)	17	10	26
21–30 years	8 (2.3%)	3	1	4
30 years +	3 (0.9%)	2	0	1
Further education in MSK pain	99 (28.9%)	27	44	28
Had LBP in past 12 months	185 (53.9%)	66	75	44
Practice setting				
Hospital	153 (44.6%)	94	59	—
Private clinic	36 (10.5%)	11	24	1
Health club	78 (22.7%)	—	6	72
Multiple locations	76 (22.2%)	18	35	23
Frequency of exercise/physical activity				
No regular exercise	113 (32.9%)	61	43	9
1 day per week	22 (6.4%)	5	15	2
2 days per week	39 (11.4%)	19	14	6
3 days per week	40 (11.7%)	15	15	10
4 days per week	39 (11.4%)	10	13	16
5 days per week	45 (13.1%)	6	11	28
6 days per week	33 (9.6%)	4	8	21
7 days per week	12 (3.5%)	3	5	4
Intensity of exercise/physical activity				
Low intensity: 1–3	143 (41.7%)	68	62	13
Moderate intensity: 4–6	158 (46.1%)	50	56	52
High intensity: 7–10	42 (12.2%)	5	6	31
Location				
Lahore	186 (53.6%)	66	63	57
Islamabad	49 (14.1%)	17	19	13
Karachi	23 (6.6%)	9	4	10
Multan	41 (11.8%)	13	24	4
Other cities	44 (12.7%)	18	14	12

*Note:* Values are presented as *n* (%).

Abbreviations: DPT = Doctor of Physical Therapy; LBP = low back pain; M.Phil = Master of Philosophy; M.S = Master of Science; MBBS = Bachelor of Medicine, Bachelor of Surgery; MSK = musculoskeletal; t‐DPT = Transitional Doctor of Physical Therapy.

Most physiotherapists held a Doctor of Physical Therapy degree (83.1%), whereas 69.1% of medical practitioners held an MBBS without postgraduate training. Exercise professionals had mixed qualifications, with 46.9% holding both bachelor's and master's degrees. About one‐third of the participants (33.5%) had 2–5 years of experience, with medical practitioners showing greater variability. A total of 28.9% had undertaken additional musculoskeletal pain training, highest among physiotherapists (35.5%). LBP was self‐reported by 53.9% in the past 12 months, with the highest prevalence among physiotherapists (60.5%).

Practice settings varied across professions: most medical practitioners (76.4%) worked in hospitals, while 75.0% of exercise professionals worked in health clubs. Regarding physical activity, 32.3% of participants (mostly exercise professionals) reported high‐intensity activity, while 32.9% (primarily medical practitioners) reported no regular exercise.

### 3.1 Group Differences in Attitudes, Imaging Beliefs, and Vignettes

The mean biomedical orientation score on the PABS‐PT was 42.5 ± 7.83 across the sample. Physiotherapists reported the highest biomedical scores (44.12 ± 8.00), while medical practitioners scored the lowest (41.51 ± 7.44), with significant between‐group differences (*p* = 0.01, *η* ^2^ = 0.02). Biopsychosocial orientation scores did not differ significantly between groups (*p* = 0.56). MSBQ scores were moderate overall (mean = 17.27 ± 3.13) with no statistically significant group differences (*p* = 0.42, *η* ^2^ = 0.005). These group‐level comparisons for PABS‐PT, MSBQ, and vignette responses are summarised in Table 2.

**TABLE 2:** Group and between‐group PABS‐PT, MSBQ and vignette responses (*p* < 0.05, small effect size *η* ^2^ ≥ 0.01).

Domain/item	Overall (*n* = 343)	Medical practitioners (*n* = 123)	Physiotherapists (*n* = 124)	Exercise professionals (*n* = 96)	*p* value	Partial eta^2^ (*η* ^2^)
PABS‐PT orientation—Biomedical						
Item 1	4.28 ± 1.11	4.24 + 1.09	4.27 ± 1.15	4.35 ± 1.08		
Item 2	4.25 ± 1.21	4.26 + 1.13	4.33 ± 1.17	4.14 ± 1.34		
Item 3	4.24 ± 1.18	4.25 + 1.10	4.37 ± 1.21	4.08 ± 1.21		
Item 4	4.56 ± 1.25	4.40 + 1.09	4.71 ± 1.28	4.58 ± 1.38		
Item 5	4.19 ± 1.29	4.08 + 1.25	4.41 ± 1.28	4.06 ± 1.32		
Item 6	4.28 ± 1.26	4.04 + 1.28	4.66 ± 1.13	4.11 ± 1.31		
Item 7	4.52 ± 1.17	4.24 + 1.27	4.72 ± 1.13	4.61 ± 1.00		
Item 8	4.27 ± 1.20	4.19 + 1.14	4.43 ± 1.10	4.18 ± 1.37		
Item 9	3.63 ± 1.28	3.69 + 1.17	3.81 ± 1.26	3.32 ± 1.41		
Item 10	4.22 ± 1.15	4.08 + 1.22	4.37 ± 1.03	4.21 ± 1.20		
Total score	42.5 ± 7.83	41.51 + 7.44	44.12 ± 8.00	41.68 ± 7.83	0.01	0.02
PABS‐PT orientation—Biopsychosocial						
Item 11	4.11 ± 1.24	4.23 ± 1.45	4.04 ± 1.20	4.06 ± 1.00		
Item 12	4.32 ± 1.29	4.49 ± 1.38	4.19 ± 1.20	4.28 ± 1.28		
Item 13	3.50 ± 1.41	3.44 ± 1.60	3.62 ± 1.21	3.40 ± 1.39		
Item 14	3.19 ± 1.42	3.25 ± 1.59	2.97 ± 1.32	3.41 ± 1.27		
Item 15	3.36 ± 1.36	3.41 ± 1.52	3.24 ± 1.25	3.44 ± 1.27		
Item 16	3.05 ± 1.42	3.04 ± 1.56	2.95 ± 1.31	3.19 ± 1.37		
Item 17	3.82 ± 1.21	3.99 ± 1.39	3.67 ± 1.05	3.81 ± 1.13		
Item 18	2.61 ± 1.39	2.52 ± 1.52	2.76 ± 1.29	2.55 ± 1.32		
Item 19	4.15 ± 1.32	4.19 ± 1.54	4.20 ± 1.04	4.02 ± 1.32		
Total score	32.15 ± 6.61	32.59 ± 7.99	31.69 ± 5.65	32.19 ± 5.80	0.56	0.003
Medical scans beliefs questionnaire						
Item 1	3.53 ± 1.01	3.56 ± 1.13	3.44 ± 0.93	3.61 ± 0.95		
Item 2	3.41 ± 0.92	3.36 ± 0.98	3.38 ± 0.84	3.52 ± 0.94		
Item 3	3.31 ± 0.94	3.39 ± 1.08	3.21 ± 0.78	3.33 ± 0.93		
Item 4	3.24 ± 0.92	3.27 ± 1.11	3.18 ± 0.74	3.27 ± 0.86		
Item 5	3.77 ± 0.99	3.78 ± 1.08	3.75 ± 0.91	3.78 ± 1.00		
Total score	17.27 ± 3.13	17.38 ± 3.62	16.99 ± 2.77	17.52 ± 2.91	0.42	0.005
Vignette # 1						
Severity	2.76 ± 0.81	2.87 ± 0.78	2.59 ± 0.79	2.85 ± 0.85	0.01	0.02
Pathology	2.22 ± 1.02	2.27 ± 1.00	1.98 ± 1.03	2.46 ± 0.97	0.002	0.03
Activity	2.70 ± 1.00	2.69 ± 1.03	2.64 ± 0.90	2.79 ± 1.08	0.55	0.003
Work	3.05 ± 0.97	3.09 ± 1.07	3.11 ± 0.83	2.92 ± 1.01	0.31	0.006
Vignette # 2						
Severity	3.52 ± 0.77	3.43 ± 0.75	3.52 ± 0.73	3.62 ± 0.82	0.20	0.009
Pathology	3.24 ± 0.88	3.12 ± 0.94	3.26 ± 0.78	3.36 ± 0.91	0.12	0.012
Activity	3.56 ± 1.08	3.65 ± 1.07	3.58 ± 0.95	3.41 ± 1.23	0.26	0.007
Work	3.88 ± 0.91	3.79 ± 1.01	4.05 ± 0.75	3.77 ± 0.94	0.03	0.02

*Note:* Values are mean ± SD. *p*‐values derived from ANOVA.

Abbreviations: *η* ^2^ = effect size (eta squared); ANOVA = analysis of variance; PABS‐PT = Pain Attitudes and Beliefs Scale for Physiotherapists.

In vignette #1, significant differences were observed for severity and pathology ratings. Medical practitioners rated severity higher than physiotherapists, whereas exercise professionals rated both severity and pathology highest. No significant differences were found for activity or work advice. In vignette #2, only work‐related recommendations differed by group, with physiotherapists rating work‐related limitations more strongly than the other groups.

### 3.2 Predictors of Clinical Decision Making

Stepwise linear regressions were used to predict vignette‐based decisions (severity, pathology, activity, and work recommendation). For vignette #1 severity ratings, years of clinical experience, exercise intensity, and education level were retained as predictors in the final model, explaining 7.1% of the variance (adjusted *R* ^2^ = 0.07, *F* = 9.73, *p* < 0.001). For pathology ratings, exercise intensity and years of experience were retained, explaining 4.5% of the variance (adjusted *R* ^2^ = 0.05, *p* < 0.001). No significant predictors were retained for activity recommendations. Gender was retained as the sole predictor of work‐related advice (adjusted *R* ^2^ = 0.03, *p* = 0.002). Regression models for vignette‐based clinical decision‐making variables are presented in Table 3.

**TABLE 3:** Stepwise linear regression models predicting clinical decision‐making for LBP vignettes.

Vignette	Outcome	Retained predictor	Standardised *β*	*p* value	Adjusted *R* ^2^	Model *F* (*p*)
1	Symptoms severity	Years of experience	0.183	0.001	0.071	9.73 (< 0.001)
		Exercise intensity	0.147	0.008		
		Education level	0.144	0.008		
1	Symptoms attributed to pathology	Exercise intensity	0.164	0.002	0.045	9.10 (< 0.001)
		Years of experience	0.130	0.016		
1	Physical activity recommendation	No variables retained	—	—	—	—
1	Work recommendation	Gender	−0.166	0.002	0.025	9.61 (0.002)
2	Symptoms	Age	0.143	0.009	0.027	5.69 (0.004)
	Severity	Gender	−0.140	0.010		
2	Symptoms attributed to pathology	Age	0.124	0.021	0.021	4.72 (0.009)
		Biomedical belief score	0.108	0.045		
2	Physical activity recommendation	Biomedical belief score	0.124	0.021	0.013	5.36 (0.021)
2	Work recommendation	Biomedical belief score	0.188	< 0.001	0.050	10.04 (< 0.001)
		Gender	−0.138	0.009		

*Note:* Forward stepwise linear regression was used. Candidate predictors entered into each model included age, gender, profession, education level, years of clinical experience, further musculoskeletal pain training, personal physical activity variables, and PABS‐PT biomedical and biopsychosocial subscale scores (see Methods). Only predictors retained in the final models are shown; all other candidate variables entered into the stepwise procedure were non‐significant and excluded. Models with no retained predictors indicate that none of the candidate variables met the entry criterion.

Abbreviations: *β* = standardised regression coefficient; adjusted *R* ^2^ = proportion of variance explained.

*p* < 0.05 considered statistically significant.

In vignette #2, age and gender were retained as predictors of severity ratings (adjusted *R* ^2^ = 0.03, *p* = 0.004). Pathology ratings were predicted by age and biomedical orientation (adjusted *R* ^2^ = 0.02, *p* = 0.009). Biomedical orientation was retained as the sole predictor of activity recommendations (adjusted *R* ^2^ = 0.01, *p* = 0.021). Work ratings were predicted by biomedical orientation and gender, together explaining 5.0% of variance (adjusted *R* ^2^ = 0.05, *F* = 10.03, *p* < 0.001). Candidate predictors entered the stepwise models are described in the Methods; variables not retained in the final models were non‐significant.

### 3.3 Predictors of Confidence in Treatment Modalities

Biopsychosocial orientation was a consistent predictor of confidence across most treatments. For over‐the‐counter medications, profession and biopsychosocial orientation explained 8.2% of the variance (adjusted *R* ^2^ = 0.08, *p* < 0.001). For prescription medications, biopsychosocial orientation and education level explained 5.0% of variance. Confidence in exercise was influenced by biopsychosocial orientation, gender, further musculoskeletal pain training, and education level (adjusted *R* ^2^ = 0.07, *p* < 0.001). For manual therapy, biopsychosocial orientation, years of experience, and gender were retained as predictors, explaining 5.7% of variance (adjusted *R* ^2^ = 0.06, *p* < 0.001). Confidence in pain education was associated with biopsychosocial orientation, education level, and exercise intensity (adjusted *R* ^2^ = 0.05, *p* < 0.001). Confidence in CBT was predicted solely by biopsychosocial orientation (adjusted *R* ^2^ = 0.03, *p* = 0.002). No significant predictors were identified for confidence in surgery. Regression models predicting confidence in each treatment modality are shown in Table 4. All candidate predictors not retained using the stepwise procedure were non‐significant. Descriptive confidence ratings for LBP treatment modalities stratified by professional group are presented in Table 5.

**TABLE 4:** Stepwise linear regression predicting healthcare professionals' confidence in LBP treatment modalities.

Treatment modality	Retained predictor	Standardised *β*	*p* value	Adjusted *R* ^2^	Model *F* (*p*)
OTC pain medication	Biopsychosocial subscale	−0.277	< 0.001	0.082	16.25 (< 0.001)
	Profession	−0.112	0.032		
Prescription pain medication	Biopsychosocial subscale	−0.183	0.001	0.050	10.08 (< 0.001)
	Education level	0.151	0.004		
Surgery	No variables retained	—	—	—	—
Exercise	Biopsychosocial subscale	−0.168	0.001	0.067	7.17 (< 0.001)
	Gender	−0.134	0.011		
	Further MSK pain education	−0.139	0.009		
	Education level	0.121	0.021		
Manual therapy	Biopsychosocial subscale	−0.152	0.004	0.057	7.92 (< 0.001)
	Years of experience	0.171	0.001		
	Gender	−0.140	0.009		
Pain education	Biopsychosocial subscale	−0.169	0.001	0.051	7.08 (< 0.001)
	Education level	0.148	0.006		
	Exercise intensity	0.143	0.009		
Cognitive behavioural therapy	Biopsychosocial subscale	−0.168	0.002	0.025	9.91 (0.002)

*Note:* Forward stepwise linear regression was used. Candidate predictors entered into each model included demographic and professional characteristics, personal physical activity variables, and PABS‐PT biomedical and biopsychosocial subscale scores (see Methods). Only predictors retained in the final models are presented; variables entered but not retained by the stepwise procedure were non‐significant. Models with no retained predictors indicate that none of the candidate variables met the entry criterion. Biopsychosocial subscale from PABS‐PT.

Abbreviations: *β* = standardised regression coefficient; adjusted *R* ^2^ = proportion of variance explained; CBT = cognitive behavioural therapy; MSK = musculoskeletal; OTC = over the counter.

**TABLE 5:** Confidence in low back pain treatment modalities by profession.

Treatment modality	Medical practitioners (mean ± SD)	Physiotherapists (mean ± SD)	Exercise professionals (mean ± SD)	Overall (mean ± SD)
Over‐the‐counter pain medication	4.30 ± 1.06	4.27 ± 0.95	4.02 ± 1.10	4.21 ± 1.04
Prescription pain medication	4.14 ± 1.11	4.12 ± 1.04	3.88 ± 1.24	4.06 ± 1.13
Surgery	3.36 ± 1.45	3.55 ± 1.08	3.26 ± 1.52	3.40 ± 1.35
Exercise	4.83 ± 1.08	4.77 ± 0.99	4.65 ± 1.26	4.76 ± 1.10
Manual therapy/massage	4.37 ± 1.37	4.56 ± 1.12	4.43 ± 1.21	4.46 ± 1.24
Pain education	4.48 ± 1.31	4.38 ± 1.08	4.49 ± 1.31	4.45 ± 1.23
Cognitive behavioural therapy	4.15 ± 1.18	4.18 ± 1.08	4.09 ± 1.19	4.15 ± 1.15

### 3.4 Predictors of Treatment Practices for LBP

Binary logistic regression models were performed to identify predictors of healthcare professionals' use of exercise and pain education as treatment options for LBP. Physiotherapists (OR = 1.70, 95% CI = 0.77–3.73, *p* = 0.189) and exercise professionals (OR = 1.11, 95% CI = 0.49–2.53, *p* = 0.801) showed no significant difference from medical practitioners. Interestingly, higher biomedical orientation was significantly associated with increased use of exercise (OR = 1.05, 95% CI = 1.01–1.10, *p* = 0.028). Physiotherapists were over five times more likely to use pain education (*β* = 1.74, *p* < 0.001, OR = 5.71, 95% CI = 3.06–10.67), whereas exercise professionals were over six times more likely (*β* = 1.79, *p* < 0.001, OR = 6.03, 95% CI = 3.22–11.32) than medical practitioners. Neither biomedical nor biopsychosocial orientation significantly predicted pain education use. Odds ratios and confidence intervals for predictors of reported use of exercise and pain education are displayed in Table 6.

**TABLE 6:** Binary logistic regression models predicting the likelihood of using exercise and pain education for LBP.

Predictor variables	Use of exercise for management of LBP				Use of pain education for management of LBP			
	*β*	*p*‐value	OR	95% CI for OR	*β*	*p*‐value	OR	95% CI for OR
Medical practitioners		0.331				0.000		
Physiotherapists versus medical practitioners	0.528	0.189	1.696	0.771, 3.730	1.742	0.000	5.711	3.058, 10.666
Exercise professionals versus medical practitioners	0.106	0.801	1.112	0.488, 2.534	1.797	0.000	6.032	3.216, 11.316
Biomedical subscale—PABS‐PT	0.050	0.028	1.051	1.005, 1.098	0.000	0.976	1.000	0.971, 1.029
Biopsychosocial subscale—PABS‐PT	0.029	0.219	1.029	0.983, 1.078	0.007	0.679	1.007	0.974, 1.042

Abbreviations: *β* = regression coefficient; CI = confidence interval; OR = odds ratio; PABS‐PT = Pain Attitudes and Beliefs Scale for Physiotherapists.

## 4 Discussion

### 4.1 Participant Characteristics

This is the first study to examine the attitudes, beliefs and confidence of Pakistani healthcare professionals regarding low back pain management. This study included 343 HCPs from Pakistan, comprising medical practitioners, physiotherapists, and exercise professionals, with distinct demographic and professional profiles. The predominance of younger physiotherapists and the overall balanced gender distribution align with the evolving trends in healthcare workforce demographics worldwide. However, gender disparities among physiotherapists (70.2% female) and exercise professionals (69.8% male) in our sample are consistent with global workforce data reported by the World Confederation for Physical Therapy for 2023 (World Physiotherapy 2024). These gender norms within these professions are reflected in South Asian countries and reported in Indian physiotherapy research on gender‐linked clinical reasoning (Bansal et al. 2024), and similarly in Spain (Pérez et al. 2022). The large proportion of physiotherapists holding a Doctor of Physical Therapy (DPT) degree underscores advancements in professional education in Pakistan (Memon et al. 2016). While such qualifications are linked with biopsychosocial reasoning in high‐income contexts such as the U.S. (Rufa et al. 2022), Brazilian data suggests that higher education may still reinforce biomedical beliefs unless curricula are modernised (L. S. Ferreira et al. 2023). Over half of the sample reported LBP in the past year, with the highest prevalence among physiotherapists (60.5%). This aligns with research highlighting the occupational risks associated with prolonged physical activities and patient handling among physiotherapists (Bryndal et al. 2022). This trend is observed globally, including in Pakistan, where pain experience may influence conservative clinical decisions (H. Y. Khan et al. 2024). Additionally, exercise professionals reported higher physical activity levels and intensity than their counterparts, supporting global evidence that personal exercise habits influence professional attitudes towards exercise interventions (Gnanendran et al. 2011). These demographic and professional factors provide important context for interpreting clinicians' clinical decision‐making patterns and highlight the role of broader cultural and educational systems in shaping care for LBP in Pakistan.

### 4.2 Attitudes and Beliefs Regarding LBP

Our findings reveal significant differences in biomedical orientation between professional groups, with physiotherapists exhibiting the strongest biomedical focus. This aligns with literature from both high‐ and low‐income settings. In Spain, physiotherapists often default to structural explanations for pain despite acknowledging biopsychosocial concepts, showing a disconnect linked to insufficient training in psychological aspects of care (Díaz‐Fernández et al. 2024). Similarly, a Brazilian survey found stronger biomedical beliefs among younger and more academically trained physiotherapists. This highlights a paradox. Advanced degrees may reinforce pathoanatomical thinking unless curricula are bio‐psychosocially enriched (M. L. Ferreira et al. 2023). The lack of significant differences in biopsychosocial orientation suggests a shared, yet limited, understanding of the psychosocial aspects of LBP among Pakistani HCPs. Similar challenges were reported in Norway and Australia, where physiotherapists struggled to meaningfully apply biopsychosocial principles due to institutional time constraints and emotionally distant training models (Dillon et al. 2023; Eland 2020).

Recent studies exploring the implementation of a biopsychosocial model in MSK care underscore how, globally, the biomedical model remains entrenched in clinical reasoning despite growing advocacy for a biopsychosocial approach (Mescouto 2023). This resistance may stem from training curricula (focused on anatomy, pathology and biomechanics), healthcare system structures, and sociocultural expectations that continue to reinforce a structural understanding of pain. In Pakistan, these barriers are compounded by limited interdisciplinary training and systemic constraints. These studies emphasise that beliefs grounded in tissue damage can foster fear‐avoidance and maladaptive patient behaviours, issues also seen in our sample, particularly among physiotherapists (Mescouto 2023). Biomedical beliefs, especially when unbalanced, can lead to passive treatment approaches and poorer outcomes (Alshehri et al. 2020).

### 4.3 Beliefs About Medical Scans and Imaging for LBP

The total Medical Scans Beliefs Questionnaire (MSBQ) scores indicated moderate agreement across all healthcare professional groups that imaging is essential for LBP care (mean = 17.27 ± 3.13). Among the items, the strongest agreement was seen for the statement ‘Medical scans are necessary to get the best medical care for low back pain’ (mean = 3.77), suggesting a persistent perception of imaging as integral to effective care. Conversely, participants expressed lower agreement with the statement that symptom changes would be directly reflected in scan findings.

There were no statistically significant differences in total MSBQ scores between medical practitioners, physiotherapists, and exercise professionals (*p* = 0.655), indicating a shared belief pattern across professional groups. These findings are similar to previous literature from LMICs, where overuse of imaging is often driven by biomedical training paradigms, limited clinical decision support tools, and patient demand for definitive diagnoses (Sharma et al. 2024; Albarqouni et al. 2022). A systematic review found that both clinicians and patients viewed imaging as crucial for ‘locating the source of pain’ and legitimising symptoms often due to fear of litigation and patient expectations (Sharma et al. 2020). Furthermore, clinician anxiety about missing serious pathology may also contribute to unnecessary imaging referrals (Sharma et al. 2024). Financial incentives and systemic pressures in the private healthcare sector may further encourage overuse of imaging in some LMIC settings (Albarqouni et al. 2022). Evidence shows that unnecessary imaging can reinforce patients' maladaptive beliefs such as fear‐avoidance and catastrophizing. It may also medicalise symptoms and undermine confidence in active self‐management strategies (Sharma et al. 2020). In many LMICs, including Pakistan, medical imaging such as MRI is often perceived as a diagnostic necessity. It is also seen as a symbol of serious, modern, and legitimate care, reinforcing public trust in structurally focused interventions regardless of clinical indication. In contrast, international clinical guidelines emphasise minimising the use of imaging for non‐specific LBP unless red flags are present (De Campos 2017).

These findings should also be interpreted in the context of patient expectations. Patients with LBP often expect diagnostic imaging and biomedical explanations, which can influence clinician decision‐making and contribute to imaging use. Inadequate management of these expectations may reinforce maladaptive beliefs and contribute to nocebo effects, potentially increasing clinician burden and frustration. Addressing patient expectations through effective communication is therefore an important component of guideline‐concordant care (Sharma et al. 2020; Colloca and Finniss 2012).

### 4.4 Influence of Attitudes, Beliefs and Associated Factors on Clinical Decision Making for LBP

Our stepwise regression analyses identified significant predictors for clinical decision‐making across vignette items. Professional experience, intensity of physical activity, and education level were significant predictors of clinician's symptom severity ratings (adjusted *R* ^2^ = 0.071, *p* < 0.001). These findings agree with data from high‐income countries (HIC) such as Australia, where increased experience is associated with reduced reliance on biomedical heuristics and greater clinical confidence in managing LBP without imaging or passive modalities (Gibbs et al. 2020). Physical activity intensity also emerged as a significant predictor across both severity and pathology ratings. Clinicians who reported higher levels of physical activity were more likely to recommend active management techniques. This outcome supports existing evidence from Australia that clinician lifestyle and behaviour shape therapeutic preferences (Gibbs and Jones 2024).

Return to Work recommendations were significantly influenced by gender and biomedical orientation (adjusted *R* ^2^ = 0.050, *p* < 0.001). This reflects similar findings in both HICs and LMICs, where female clinicians and those with strong biomedical beliefs tend to offer more cautious return‐to‐work advice (Gibbs et al. 2022; M. L. Ferreira et al. 2023). Biomedical orientation shaping activity and pathology decisions highlight the persistent dominance of structural pain models, especially in LMICs with limited interdisciplinary training.

### 4.5 Confidence in Treatment Modalities

Confidence in treatment options varied significantly between professional groups. Physiotherapists and exercise professionals reported higher confidence in non‐pharmacological approaches such as pain education and exercise. In contrast, medical practitioners exhibited lower confidence in these modalities, reflecting discipline‐specific training and clinical exposure. Consistently, biopsychosocial orientation emerged as a significant predictor of confidence across most evidence‐based treatments, including exercise, pain education, and cognitive behavioural therapy (CBT). These findings mirror trends in HICs. For instance, Gibbs et al. (2023) found that Australian clinicians with stronger biopsychosocial beliefs demonstrated higher confidence in recommending CBT and active self‐management strategies. Similarly, a recent systematic review reported that targeted biopsychosocial education improved clinicians' confidence and willingness to use non‐pharmacological interventions for chronic pain (Mankelow et al. 2021).

In LMICs, available evidence shows similar patterns but remains limited (M. L. Ferreira et al. 2023). Most studies focus narrowly on physiotherapists, with minimal insight into cross‐disciplinary training or behavioural interventions. This highlights a significant research and implementation gap, particularly in Pakistan, where biopsychosocial competencies remain underdeveloped across healthcare sectors.

### 4.6 Use of Pain Education and Exercise for LBP Management

Logistic regression showed that physiotherapists and exercise professionals were more likely than medical practitioners to use pain education, reflecting training in movement and communication. Interestingly, higher biomedical orientation was associated with greater reported use of exercise. This suggests that, in some cases, exercise may still be applied through a biomechanical lens. This supports earlier findings that the mere use of exercise does not guarantee a biopsychosocial framing of care. A recent synthesis of literature concluded that combining therapeutic exercise with pain education is moderately effective for reducing pain and disability in chronic LBP patients (Jones et al. 2020). Similarly, in the U.S., Carey and Freburger (2016) highlighted underutilisation of exercise‐based care by physicians, despite its preventive benefits.

### 4.7 Implications and Future Directions

These findings underscore the need for interventions that transcend conceptual awareness and focus on the practical application of biopsychosocial models. Evidence suggests that multifaceted implementation strategies such as combining continuing education, peer support, decision aids, and guideline reinforcement are more effective than passive dissemination alone (Bekkering 2005; Jenkins et al. 2018). Patient booklets, when paired with provider training, have been shown to reduce unnecessary imaging and sick leave (Simula et al. 2021). Serious games, digital learning tools, and institutional restructuring (e.g., team‐based care models, incentivised guideline adherence) may further reinforce lasting behavioural change (Koelewijn et al. 2024; Suman et al. 2016).

Future research should examine whether biopsychosocial training can produce sustained shifts in clinician behaviour and patient outcomes in low‐resource contexts. Randomised trials and implementation studies using behavioural science frameworks could help identify local barriers and tailor interventions to Pakistan's health system. Crucially, interventions must not only address knowledge gaps but also challenge the persistent biomedical narratives embedded in education, clinical hierarchies, and public expectations.

### 4.8 Strengths and Limitations

A key strength of this study is its inclusion of diverse health​ professional groups, offering a holistic perspective on LBP management in Pakistan. The use of validated tools, such as PABS‐PT and MSBQ, ensures reliability and comparability with international research. Several limitations warrant consideration. The cross‐sectional design limits causal interpretations. The reliance on self‐reported, internet‐based survey data introduces the potential for response bias. Moreover, under‐representation of rural healthcare professionals may limit the generalisability of findings to less urbanised contexts.

## 5 Conclusions

This study identified key professional differences in how Pakistani healthcare providers approach low back pain. Physiotherapists demonstrated the strongest biomedical orientation. Although biopsychosocial beliefs were associated with greater confidence in non‐pharmacological treatments, these beliefs did not consistently translate into clinical practice. Beliefs about imaging were uniformly high across professions, reflecting entrenched educational and systemic biases. Clinical decisions were influenced by clinician experience, gender, physical activity, and belief orientation. This underscores the interplay between personal and professional factors. To enhance LBP care in Pakistan and similar settings, educational reforms are needed that integrate biopsychosocial reasoning, patient communication skills, and interdisciplinary training into routine healthcare practice.

## Author Contributions

Study concept and design: Muhammad Naseeb Ullah Khan. Acquisition of data: Muhammad Naseeb Ullah Khan. Analysis and interpretation of data: Muhammad Naseeb Ullah Khan. Drafting and review of the manuscript: Muhammad Naseeb Ullah Khan, Aastha Malhotra, and Melainie Cameron. Study supervision: Melainie Cameron and Aastha Malhotra.

## Ethics Statement

This study was approved by the Human Research Ethics Committee of the University of Southern Queensland (approval # ETH2023‐0010).

## Conflicts of Interest

The authors declare no conflicts of interest.

## Data Availability

The datasets used and/or analysed during the current study are available from the corresponding author on reasonable request.

## References

[msc70228-bib-0001] Albarqouni, L. , M. Arab‐Zozani , E. Abukmail , et al. 2022. “Overdiagnosis and Overuse of Diagnostic and Screening Tests in Low‐Income and Middle‐Income Countries: A Scoping Review.” BMJ Global Health 7, no. 10: e008696. 10.1136/bmjgh-2022-008696. PMC9442491 36316027

[msc70228-bib-0002] Alshehri, M. A. , H. Alzahrani , M. Alotaibi , A. Alhowimel , and O. Khoja . 2020. “Physiotherapists’ Pain Attitudes and Beliefs Towards Chronic Low Back Pain and Their Association With Treatment Selection: A Cross‐Sectional Study.” BMJ Open 10, no. 6: e037159. 10.1136/bmjopen-2020-037159. PMC7311013 32571864

[msc70228-bib-0003] Anema, J. R. , A. J. M. Schellart , J. D. Cassidy , P. Loisel , T. J. Veerman , and A. J. Van Der Beek . 2009. “Can Cross Country Differences in Return‐to‐Work After Chronic Occupational Back Pain Be Explained? An Exploratory Analysis on Disability Policies in a Six Country Cohort Study.” Journal of Occupational Rehabilitation 19, no. 4: 419–426. 10.1007/s10926-009-9202-3. 19760488 PMC2775112

[msc70228-bib-0004] Bansal, N. , P. C. Sharma , K. Bansal , and R. K. Parasher . 2024. “A Study Evaluating Attitudes Toward Treatment of Low Back Pain Among Indian Physiotherapists.” Journal of Datta Meghe Institute of Medical Sciences University 19, no. 4: 693–697. 10.4103/jdmimsu.jdmimsu_713_23.

[msc70228-bib-0005] Beales, D. , P. Kent , M. B. Birkrem , et al. 2021. “Only One Fifth of Young Australian Adults Have Beliefs About Medical Imaging for Low Back Pain That Align With Current Evidence: A Cross‐Sectional Study.” Musculoskeletal Science and Practice 56: 102460. 10.1016/j.msksp.2021.102460. 34547611

[msc70228-bib-0006] Bekkering, G. E. 2005. “Effect on the Process of Care of an Active Strategy to Implement Clinical Guidelines on Physiotherapy for Low Back Pain: A Cluster Randomised Controlled Trial.” BMJ Quality and Safety 14, no. 2: 107–112. 10.1136/qshc.2003.009357. PMC1743983 15805455

[msc70228-bib-0007] Bishop, A. , N. E. Foster , E. Thomas , and E. M. Hay . 2008. “How Does the Self‐Reported Clinical Management of Patients With Low Back Pain Relate to the Attitudes and Beliefs of Health Care Practitioners? A Survey of UK General Practitioners and Physiotherapists.” Pain 135, no. 1: 187–195. 10.1016/j.pain.2007.11.010. 18206309 PMC2258319

[msc70228-bib-0008] Bryndal, A. , S. Glowinski , and A. Grochulska . 2022. “Influence of Occupation on the Prevalence of Spinal Pain Among Physiotherapists and Nurses.” Journal of Clinical Medicine 11, no. 19: 5600. 10.3390/jcm11195600. 36233474 PMC9571452

[msc70228-bib-0009] Buchbinder, R. , F. M. Blyth , L. M. March , P. Brooks , A. D. Woolf , and D. G. Hoy . 2013. “Placing the Global Burden of Low Back Pain in Context.” Best Practice & Research Clinical Rheumatology 27, no. 5: 575–589. 10.1016/j.berh.2013.10.007. 24315140

[msc70228-bib-0010] Buchbinder, R. , M. Van Tulder , B. Öberg , et al. 2018. “Low Back Pain: A Call for Action.” Lancet 391, no. 10137: 2384–2388. 10.1016/s0140-6736(18)30488-4. 29573871

[msc70228-bib-0011] Carey, T. S. , and J. K. Freburger . 2016. “Exercise and the Prevention of Low Back Pain.” JAMA Internal Medicine 176, no. 2: 208. 10.1001/jamainternmed.2015.7636. 26752108

[msc70228-bib-0012] Colloca, L. , and D. Finniss . 2012. “Nocebo Effects, Patient‐Clinician Communication, and Therapeutic Outcomes.” JAMA 307, no. 6: 567–568. 10.1001/jama.2012.115. 22318275 PMC6909539

[msc70228-bib-0013] Darlow, B. , B. Fullen , S. Dean , D. Hurley , G. Baxter , and A. Dowell . 2011. “The Association Between Health Care Professional Attitudes and Beliefs and the Attitudes and Beliefs, Clinical Management, and Outcomes of Patients With Low Back Pain: A Systematic Review.” European Journal of Pain 16, no. 1: 3–17. 10.1016/j.ejpain.2011.06.006. 21719329

[msc70228-bib-0014] De Campos, T. F. 2017. “Low Back Pain and Sciatica in Over 16s: Assessment and Management NICE Guideline [NG59].” Journal of Physiotherapy 63, no. 2: 120. 10.1016/j.jphys.2017.02.012. 28325480

[msc70228-bib-0015] Díaz‐Fernández, Á. , I. Cortés‐Pérez , E. Obrero‐Gaitán , et al. 2024. “Chronic Pain Management Approaches Among Spanish Physiotherapists: Influences, Practices, Barriers, and Challenges.” Journal of Personalized Medicine 14, no. 9: 903. 10.3390/jpm14090903. 39338157 PMC11433413

[msc70228-bib-0016] Dillon, M. , R. Olson , K. Mescouto , N. Costa , and J. Setchell . 2023. “How Physiotherapists Attend to the Human Aspects of Care When Working With People With Low Back Pain: A Thematic Analysis.” Health Sociology Review 32, no. 3: 277–293. 10.1080/14461242.2022.2161927. 36632019

[msc70228-bib-0017] Eland, N. D. 2020. “The Attitudes and Beliefs of Physiotherapists Treating Back Pain.” PhD thesis, University of Bergen. https://bora.uib.no/bora‐xmlui/bitstream/handle/1956/24067/archive.pdf.

[msc70228-bib-0018] Eland, N. D. , A. Kvåle , R. W. J. G. Ostelo , H. C. W. De Vet , and L. I. Strand . 2018. “Discriminative Validity of the Pain Attitudes and Beliefs Scale for Physical Therapists.” Physical Therapy 99, no. 3: 339–353. 10.1093/ptj/pzy139. 30690547

[msc70228-bib-0019] Fatoye, F. , T. Gebrye , C. E. Mbada , and U. Useh . 2023. “Clinical and Economic Burden of Low Back Pain in Low‐ and Middle‐Income Countries: A Systematic Review.” BMJ Open 13, no. 4: e064119. 10.1136/bmjopen-2022-064119. PMC10151982 37185180

[msc70228-bib-0020] Faul, F. , E. Erdfelder , A. Buchner , and A. Lang . 2009. “Statistical Power Analyses Using G*Power 3.1: Tests for Correlation and Regression Analyses.” Behavior Research Methods 41, no. 4: 1149–1160. 10.3758/brm.41.4.1149. 19897823

[msc70228-bib-0021] Ferreira, L. S. , M. P. E. Silva , B. T. Saragiotto , and M. O. Magalhães . 2023. “Attitudes and Beliefs of Brazilian Physical Therapists About Chronic Nonspecific Low Back Pain and Its Impact on Clinical Decision‐Making: An Online Survey Study.” Musculoskeletal Science and Practice 67: 102832. 10.1016/j.msksp.2023.102832. 37506584

[msc70228-bib-0022] Ferreira, M. L. , K. De Luca , L. M. Haile , et al. 2023. “Global, Regional, and National Burden of Low Back Pain, 1990–2020, Its Attributable Risk Factors, and Projections to 2050: A Systematic Analysis of the Global Burden of Disease Study 2021.” Lancet Rheumatology 5, no. 6: e316–e329. 10.1016/s2665-9913(23)00098-x. 37273833 PMC10234592

[msc70228-bib-0023] Finance Division, Government of Pakistan . 2024. “Pakistan’s Total Health Expenditures.” https://www.finance.gov.pk/survey/chapter_24/11_health.pdf.

[msc70228-bib-0024] Fitzgerald, K. , B. Vaughan , M. Fleischmann , and P. Austin . 2020. “Pain Knowledge, Attitudes and Beliefs of Australian Osteopaths Drawn From a Nationally Representative Sample of the Profession.” Journal of Bodywork and Movement Therapies 24, no. 4: 43–50. 10.1016/j.jbmt.2020.06.022. 33218544

[msc70228-bib-0025] Foster, N. E. , J. R. Anema , D. Cherkin , et al. 2018. “Prevention and Treatment of Low Back Pain: Evidence, Challenges, and Promising Directions.” Lancet 391, no. 10137: 2368–2383. 10.1016/s0140-6736(18)30489-6. 29573872

[msc70228-bib-0026] Gibbs, M. T. , and M. D. Jones . 2024. “Are the Attitudes and Beliefs of Australian Exercise‐Based Practitioners Associated With Their Use of, and Confidence in, Treatment Modalities for People With Chronic Low Back Pain?” Supplement, Journal of Clinical Exercise Physiology 13, no. s2: 323. 10.31189/2165-7629-13-s2.323. 38054520

[msc70228-bib-0027] Gibbs, M. T. , T. Last , P. Marshall , and M. D. Jones . 2023. “Are the Attitudes and Beliefs of Australian Exercise‐Based Practitioners Associated With Their Use of, and Confidence in, Treatment Modalities for People With Chronic Low Back Pain?” Musculoskeletal Care 22, no. 1: e1852. 10.1002/msc.1852. 38054520

[msc70228-bib-0028] Gibbs, M. T. , N. M. Morrison , and P. W. Marshall . 2020. “Biomedical Beliefs Explain the Clinical Decisions Made by Exercise‐Based Practitioners for People With Chronic Low Back Pain.” Spine 46, no. 2: 114–121. 10.1097/brs.0000000000003698. 32947498

[msc70228-bib-0029] Gibbs, M. T. , N. M. Morrison , and P. W. Marshall . 2022. “Education Improves Decision‐Making of Exercise Physiologists Regarding Low Back Pain.” Journal of Clinical Exercise Physiology 11, no. 1: 12–18. 10.31189/2165-6193-11.1.12.

[msc70228-bib-0030] Gnanendran, A. , D. B. Pyne , K. E. Fallon , and P. A. Fricker . 2011. “Attitudes of Medical Students, Clinicians and Sports Scientists Towards Exercise Counselling.” Journal of Sports Science and Medicine 10, no. 3: 426. https://doaj.org/article/551c3416fdeb4b29a180f49667e69694. 24150613 PMC3737811

[msc70228-bib-0031] Hanif, Q. A. M. , N. A. Pradhan , W. Hameed , B. Zafar , and B. M. H. Khowaja . 2025. “Insights Into the Provision of Physical Therapy Services at Public Sector Hospitals in Pakistan: An Exploratory Qualitative Study.” BMJ Open 15, no. 4: e084854. 10.1136/bmjopen-2024-084854. PMC11969620 40180379

[msc70228-bib-0032] Heltberg, R. , and N. Lund . 2009. “Shocks, Coping, and Outcomes for Pakistan’s Poor: Health Risks Predominate.” Journal of Development Studies 45, no. 6: 889–910. 10.1080/00220380902802214.

[msc70228-bib-0033] Houben, R. M. , R. W. Ostelo , J. W. Vlaeyen , P. M. Wolters , M. Peters , and S. G. S. D. Berg . 2004. “Health Care Providers’ Orientations Towards Common Low Back Pain Predict Perceived Harmfulness of Physical Activities and Recommendations Regarding Return to Normal Activity.” European Journal of Pain 9, no. 2: 173–183. 10.1016/j.ejpain.2004.05.002. 15737810

[msc70228-bib-0034] Innes, S. I. , P. D. Werth , P. J. Tuchin , and P. L. Graham . 2015. “Attitudes and Beliefs of Australian Chiropractors’ About Managing Back Pain: A Cross‐Sectional Study.” Chiropractic & Manual Therapies 23, no. 1: 17. 10.1186/s12998-015-0062-y. 26085924 PMC4470072

[msc70228-bib-0035] Javed, A. , M. S. Khan , A. Nasar , and A. Rasheed . 2020. “Mental Healthcare in Pakistan.” Taiwanese Journal of Psychiatry 34, no. 1: 6. 10.4103/tpsy.tpsy_8_20.

[msc70228-bib-0036] Jenkins, H. J. , N. A. Moloney , S. D. French , et al. 2018. “Using Behaviour Change Theory and Preliminary Testing to Develop an Implementation Intervention to Reduce Imaging for Low Back Pain.” BMC Health Services Research 18, no. 1: 734. 10.1186/s12913-018-3526-7. 30249241 PMC6154885

[msc70228-bib-0037] Jones, K. C. , E. C. Tocco , A. N. Marshall , T. C. V. McLeod , and C. E. W. Bacon . 2020. “Pain Education With Therapeutic Exercise in Chronic Nonspecific Low Back Pain Rehabilitation: A Critically Appraised Topic.” Journal of Sport Rehabilitation 29, no. 8: 1204–1209. 10.1123/jsr.2019-0345. 32106086

[msc70228-bib-0038] Khan, H. Y. , S. H. Khan , A. Gillani , et al. 2024. “Knowledge, Attitudes and Beliefs of Clinical Physiotherapists Towards Chronic Back Pain.” National Journal of Life and Health Sciences 3, no. 1: 20–24. 10.62746/njlhs.v3n1.21.

[msc70228-bib-0039] Khan, M. N. U. , A. Malhotra , and M. Cameron . 2025. “Low Back Pain in Pakistan: A Scoping Review of Epidemiology and Treatment Approaches.” Journal of Asian Scientific Research 15, no. 4: 898–911. 10.55493/5003.v15i4.5775.

[msc70228-bib-0040] Khan, M. N. U. , N. M. Morrison , and P. W. Marshall . 2020. “The Role of Fear‐Avoidance Beliefs on Low Back Pain‐Related Disability in a Developing Socioeconomic and Conservative Culture: A Cross‐Sectional Study of a Pakistani Population.” Journal of Pain Research 13: 2377–2387. 10.2147/jpr.s258314. 33061553 PMC7520149

[msc70228-bib-0041] Khan, S. A. 2019. “Situation Analysis of Health Care System of Pakistan: Post 18 Amendments.” Health Care Current Reviews 7, no. 3. 10.35248/2375-4273.19.07.244.

[msc70228-bib-0042] Khan, S. J. , M. Asif , S. Aslam , W. J. Khan , and S. A. Hamza . 2023. “Pakistan’s Healthcare System: A Review of Major Challenges and the First Comprehensive Universal Health Coverage Initiative.” Cureus 15, no. 9: e44641. 10.7759/cureus.44641. 37799252 PMC10548490

[msc70228-bib-0043] Khan, S. U. , and I. Hussain . 2020. “Inequalities in Health and Health‐Related Indicators: A Spatial Geographic Analysis of Pakistan.” BMC Public Health 20, no. 1: 1800. 10.1186/s12889-020-09870-4. 33243192 PMC7690118

[msc70228-bib-0044] Koelewijn, G. , M. P. Hennus , H. S. M. Kort , J. Frenkel , and T. Van Houwelingen . 2024. “Games to Support Teaching Clinical Reasoning in Health Professions Education: A Scoping Review.” Medical Education Online 29, no. 1: 2316971. 10.1080/10872981.2024.2316971. 38394053 PMC10896137

[msc70228-bib-0045] Maher, C. , M. Underwood , and R. Buchbinder . 2016. “Non‐Specific Low Back Pain.” Lancet 389, no. 10070: 736–747. 10.1016/s0140-6736(16)30970-9. 27745712

[msc70228-bib-0046] Mankelow, J. , C. Ryan , P. Taylor , G. Atkinson , and D. Martin . 2021. “A Systematic Review and Meta‐Analysis of the Effects of Biopsychosocial Pain Education Upon Health Care Professional Pain Attitudes, Knowledge, Behavior and Patient Outcomes.” Journal of Pain 23, no. 1: 1–24. 10.1016/j.jpain.2021.06.010. 34237464

[msc70228-bib-0047] Memon, A. R. , N. M. Sahibzada , M. E. Azim , and F. A. Siddiqui . 2016. “Physical Therapy as a Profession and Its Educational Development in Pakistan.” JPMA. The Journal of the Pakistan Medical Association 66, no. 11: 1472–1474. https://pubmed.ncbi.nlm.nih.gov/27812071. 27812071

[msc70228-bib-0048] Mescouto, 2023 Mescouto, K. A. 2023. Enhancing Low Back Pain Care: Thinking and Practising Critically Beyond the Biopsychosocial Model. 10.14264/fb040ca.

[msc70228-bib-0049] Morriss, W. , and C. Roques . 2018. “Pain Management in Low‐ and Middle‐Income Countries.” BJA Education 18, no. 9: 265–270. 10.1016/j.bjae.2018.05.006. 33456843 PMC7807826

[msc70228-bib-0050] Mutsaers, J. , R. Peters , A. Pool‐Goudzwaard , B. Koes , and A. Verhagen . 2012. “Psychometric Properties of the Pain Attitudes and Beliefs Scale for Physiotherapists: A Systematic Review.” Manual Therapy 17, no. 3: 213–218. 10.1016/j.math.2011.12.010. 22277324

[msc70228-bib-0051] Naidu, T. 2023. “Coloniality Lives on Through Medical Education.” BMJ: p2294. 10.1136/bmj.p2294. 37844932

[msc70228-bib-0052] Oliveira, C. B. , C. G. Maher , R. Z. Pinto , et al. 2018. “Clinical Practice Guidelines for the Management of Non‐Specific Low Back Pain in Primary Care: An Updated Overview.” European Spine Journal 27, no. 11: 2791–2803. 10.1007/s00586-018-5673-2. 29971708

[msc70228-bib-0053] Peacock, S. , and S. Patel . 2008. “Cultural Influences on Pain.” Reviews in Pain 1, no. 2: 6–9. 10.1177/204946370800100203. 26525084 PMC4589930

[msc70228-bib-0054] Pérez, S. E. M. , L. L. González , I. A. Acevedo , et al. 2022. “Attitudes and Beliefs Towards Low Back Pain (LBP) Among Physiotherapists in Spain.” Bulletin of Faculty of Physical Therapy 27, no. 1: 52. 10.1186/s43161-022-00112-9.

[msc70228-bib-0055] Protheroe, E. 2024. “Overuse of Medical Imaging in Low‐Middle Income Countries: A Scoping Review.” Journal of Global Radiology 10, no. 1. 10.7191/jgr.906.

[msc70228-bib-0056] Rainville, J. , N. Carlson , P. Polatin , R. J. Gatchel , and A. Indahl . 2000. “Exploration of Physicians’ Recommendations for Activities in Chronic Low Back Pain.” Spine 25, no. 17: 2210–2220. 10.1097/00007632-200009010-00012. 10973405

[msc70228-bib-0057] Rufa, A. , M. Dolphin , K. Adams , and G. Brooks . 2022. “Factors Associated With the Low Back Pain‐Related Attitudes and Beliefs of Physical Therapists.” Musculoskeletal Science and Practice 58: 102518. 10.1016/j.msksp.2022.102518. 35131592

[msc70228-bib-0058] Sharma, S. , A. Pathak , R. Parker , et al. 2024. “How Low Back Pain Is Managed—A Mixed‐Methods Study in 32 Countries. Part 2 of Low Back Pain in Low‐ and Middle‐Income Countries Series.” Journal of Orthopaedic and Sports Physical Therapy 54, no. 8: 560–572. 10.2519/jospt.2024.12406. 38602844

[msc70228-bib-0059] Sharma, S. , A. C. Traeger , B. Reed , et al. 2020. “Clinician and Patient Beliefs About Diagnostic Imaging for Low Back Pain: A Systematic Qualitative Evidence Synthesis.” BMJ Open 10, no. 8: e037820. 10.1136/bmjopen-2020-037820. PMC7451538 32830105

[msc70228-bib-0060] Simula, A. S. , H. J. Jenkins , M. J. Hancock , A. Malmivaara , N. Booth , and J. Karppinen . 2021. “Patient Education Booklet to Support Evidence‐Based Low Back Pain Care in Primary Care – A Cluster Randomized Controlled Trial.” BMC Family Practice 22, no. 1: 178. 10.1186/s12875-021-01529-2. 34493219 PMC8422671

[msc70228-bib-0061] Slade, S. C. , P. Kent , S. Patel , T. Bucknall , and R. Buchbinder . 2015. “Barriers to Primary Care Clinician Adherence to Clinical Guidelines for the Management of Low Back Pain.” Clinical Journal of Pain 32, no. 9: 800–816. 10.1097/ajp.0000000000000324. 26710217

[msc70228-bib-0062] Sohaib, A. , M. Hassan , M. Hussain , and A. Hannan . 2025. “Socioeconomic and Geographical Disparities in Healthcare Quality in Pakistan.” Journal of the Pakistan Medical Association 74, no. 6: 1031. 10.47391/jpma.21573. 40698498

[msc70228-bib-0063] Suman, A. , F. G. Schaafsma , R. Buchbinder , M. W. Van Tulder , and J. R. Anema . 2016. “Implementation of a Multidisciplinary Guideline for Low Back Pain: Process‐Evaluation Among Health Care Professionals.” Journal of Occupational Rehabilitation 27, no. 3: 422–433. 10.1007/s10926-016-9673-y. PMC5591342 27699618

[msc70228-bib-0064] Traeger, A. , R. Buchbinder , A. Elshaug , P. Croft , and C. Maher . 2019. “Care for Low Back Pain: Can Health Systems Deliver?” Bulletin of the World Health Organization 97, no. 6: 423–433. 10.2471/blt.18.226050. 31210680 PMC6560373

[msc70228-bib-0065] Williams, J. S. , N. Ng , K. Peltzer , et al. 2015. “Risk Factors and Disability Associated With Low Back Pain in Older Adults in Low‐ and Middle‐Income Countries. Results From the WHO Study on Global AGEing and Adult Health (SAGE).” PLoS One 10, no. 6: e0127880. 10.1371/journal.pone.0127880. 26042785 PMC4456393

[msc70228-bib-0066] World Physiotherapy . 2024. “World Confederation for Physical Therapy.” Annual Membership Census 2023 – Pakistan. https://world.physio/sites/default/files/2024‐01/AMC2023‐Pakistan.pdf.

[msc70228-bib-0067] Wu, A. , L. March , X. Zheng , et al. 2020. “Global Low Back Pain Prevalence and Years Lived With Disability From 1990 to 2017: Estimates From the Global Burden of Disease Study 2017.” Annals of Translational Medicine 8, no. 6: 299. 10.21037/atm.2020.02.175. 32355743 PMC7186678

[msc70228-bib-0068] Zadro, J. , M. O’Keeffe , and C. Maher . 2019. “Do Physical Therapists Follow Evidence‐Based Guidelines When Managing Musculoskeletal Conditions? Systematic Review.” BMJ Open 9, no. 10: e032329. 10.1136/bmjopen-2019-032329. PMC6797428 31591090

[msc70228-bib-0069] Zhou, T. , D. Salman , and A. H. McGregor . 2024. “Recent Clinical Practice Guidelines for the Management of Low Back Pain: A Global Comparison.” BMC Musculoskeletal Disorders 25, no. 1: 344. 10.1186/s12891-024-07468-0. 38693474 PMC11061926

